# Impact of Pt grain size on ferroelectric properties of zirconium hafnium oxide by chemical solution deposition

**DOI:** 10.1186/s40580-022-00334-6

**Published:** 2022-10-05

**Authors:** An Hoang-Thuy Nguyen, Manh-Cuong Nguyen, Anh-Duy Nguyen, Ji-Yong Yim, Jeong-Han Kim, No-Hwal Park, Seung-Joon Jeon, Daewoong Kwon, Rino Choi

**Affiliations:** 1 Department of Materials Science and Engineering, Inha University, Incheon, 22212 South Korea; 2 Department of Electrical Engineering at, Inha University, Incheon, 22212 South Korea; 3 3D Convergence Center at Inha University, Incheon, 22212 South Korea

**Keywords:** Chemical solution deposition, Hafnium zirconium oxide, Pt bottom electrodes, Phase transformation, Pt grain size

## Abstract

The effects of the grain size of Pt bottom electrodes on the ferroelectricity of hafnium zirconium oxide (HZO) were studied in terms of the orthorhombic phase transformation. HZO thin films were deposited by chemical solution deposition on the Pt bottom electrodes with various grain sizes which had been deposited by direct current sputtering. All the samples were crystallized by rapid thermal annealing at 700 °C to allow a phase transformation. The crystallographic phases were determined by grazing incidence X-ray diffraction, which showed that the bottom electrode with smaller Pt grains resulted in a larger orthorhombic phase composition in the HZO film. As a result, capacitors with smaller Pt grains for the bottom electrode showed greater ferroelectric polarization. The smaller grains produced larger in-plane stress which led to more orthorhombic phase transformation and higher ferroelectric polarization.

## Introduction

Materials based on hafnium oxide thin films have attracted considerable interest owing to their compatibility with conventional device fabrication processes and potential applications, such as ferroelectric field-effect-transistors, ferroelectric random access memory, and ferroelectric tunneling junction [1–3]. Further applications of hafnium oxide-based ferroelectric thin films include energy-related applications such as pyroelectric energy harvesting, electrocaloric cooling, and electrostatic energy storage [4–6]. The ferroelectricity was attributed to the non-centrosymmetric orthorhombic phase with a Pca2_1_ space group [7]. Achieving the orthorhombic phase in hafnium oxide thin films depends on the post-annealing treatment [8], growth temperature [9], surface energy [10], thicknesses [11], mechanical stress from top electrodes [12], and dopants [13]. Besides, zirconium is a favored dopant species among the doping elements because of its similar atomic size. Zirconium doping into hafnium oxide enlarges a window for realizing good ferroelectric properties such as stable ferroelectricity, low annealing temperature, and high doped percentage (up to 50 mol %). Thus far, several studies have achieved stable and polarized zirconium doped hafnium oxide (HZO) thin films using various processes.

Since the ferroelectric properties of hafnium-based oxide were first reported [14], there has been considerable debate regarding the physical origin of the ferroelectric properties and the critical conditions. On the other hand, polarization hysteresis is observed in HZO thin films deposited by many methods, such as atomic layer deposition (ALD) [15], pulsed laser deposition [16], co-sputtering deposition [17], and chemical solution deposition (CSD) [18]. Among those procedures, CSD can be an opportunity because it is a low-cost and straightforward process, allows the addition of various dopants, and exhibits minor thickness effects on ferroelectric properties [13, 19, 20]. In addition, CSD can extend the window of the deposition conditions by tailoring the solution properties [21]. The CSD HZO thin film is dependent on the morphology of the substrate, on which they are deposited. Thus, the condition of the substrates has significant effects on the crystalline structure and ferroelectric properties of the CSD HZO thin film. In other hand, to determine the influence of substrates on the ferroelectric properties by the ALD process, several bottom electrodes such as Si, SiGe, Ge, and Pt beside standard TiN have been studied in terms of their electrical, material, and reliability characteristics [22, 23]. The change in ferroelectricity is explained by the thermal expansion coefficient of bottom electrode materials. Based on the results, the strain effects attributed to the different thermal expansion coefficients (TEC) are important factors that affect the phase transformation. One of the most crucial factors to determine the TEC of the substrate is the grain size. Nevertheless, the effects of grain size of the substrate on the ferroelectric properties of HZO have received little attention.

This study examined the effects of the grain size of Pt electrodes on the ferroelectric properties. The grain size of the Pt bottom electrodes was first controlled by the deposition temperatures during the sputtering process. HZO films were then deposited by CSD using chloride precursors for the phase transformation process. The electrical effects of the bottom electrodes were extracted from the polarization-electrical field (P-E) and capacitance-electrical field (C-E) performances.

## Experimental

Pt bottom electrodes, 100 nm thickness, were deposited by direct current (DC) sputtering on Si/SiO_2_ (300 nm) with a 10 nm thick Ti interlayer as an adhesion layer. The Pt grain sizes were varied by changing the substrate temperature during deposition: room temperature (RT), 200 °C, or 400 °C. ZrO_2_ and HfO_2_ precursors were fabricated by dissolving zirconium chloride (ZrCl_4_, Sigma Aldrich, 99.9%) and hafnium chloride (HfCl_4_, Alfa Aesar, 99.9%) powders separately in 2-methoxyethanol (2-ME, Sigma Aldrich, 99.9%). The two solutions were stirred separately at 60 °C for three hours until both became clear and transparent. Subsequently, the 0.2 M HZO solution was formed by mixing the Hf and Zr solutions with a ratio of 7:3, followed by stirring for six hours at 60 °C. The HZO solution was then aged at room temperature for at least 24 h before spin coating.

**Fig. 1:**
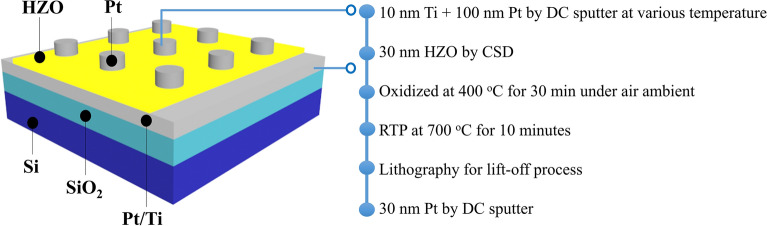
MFM structure with HZO thin film on Pt substrates with various grain sizes and the process

The metal-ferroelectric-metal (MFM) structure was fabricated, as shown in Fig. 1. The HZO solution was coated on all Si/SiO_2_/Ti/Pt substrates by spin coating at 3000 rpm for 40 s and baking at 150 °C for 90 s. Precursor preparation and spin coating were performed inside a glove box to eliminate the effects of humidity. The samples were then oxidized at 400 °C for 30 min under air ambient. Due to the TEC differences, the Pt top electrodes were peeled off after the annealing process. Thus all the samples were annealed with post-deposition annealing. The HZO thin films were crystallized at an optimum temperature of 700 °C for 10 min by RTA in an N_2_ environment to obtain the orthorhombic phase for the ferroelectric properties. Finally, 50 nm Pt top electrodes were deposited at room temperature by DC sputtering and patterned using a lift-off process.

Scanning electron microscopy (SEM) and atomic force microscopy (AFM) were used to characterize the grain size and surface morphology of the Pt thin films. The thickness of the annealed-HZO thin films was determined by alpha-step D-500. The crystalline structure of HZO layers was examined by grazing incidence X-ray diffraction (GIXRD). The electrical properties of the HZO films on all the Pt substrates were measured using a Keithley 4200A-SCS parameter analyzer in a dark chamber at room temperature.

## Results and discussion

**Fig. 2:**
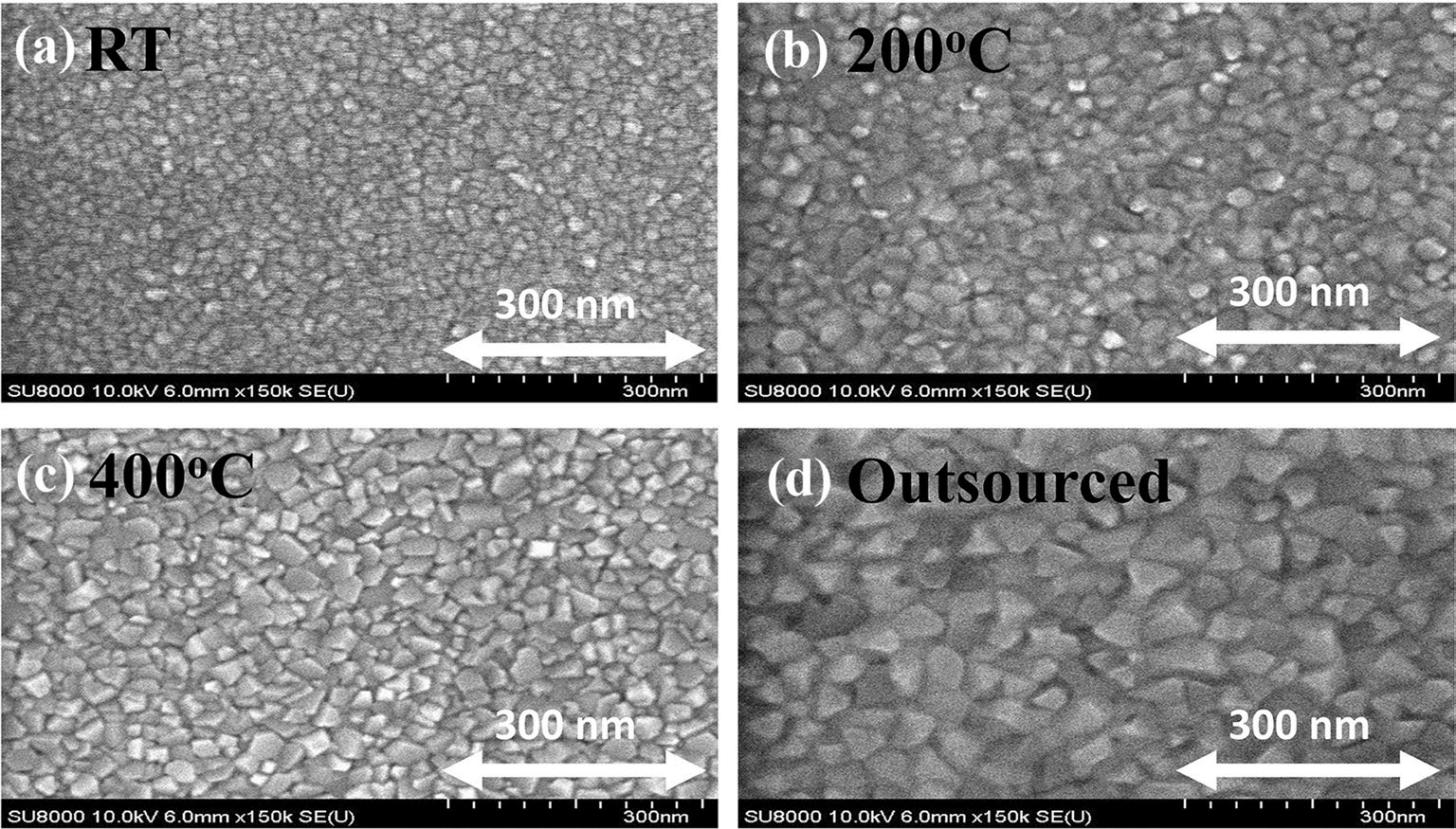
Surface SEM images of Pt films with various deposition temperatures as **a** room temperature, **b** 200 °C, **c** 400 °C, and **d** outsourced samples

The principle effect in this work is the different grain sizes of the Pt layers for the bottom electrodes, as shown in the SEM images in Fig. 2. The grain size was controlled by varying the deposition temperature with the same power, environment, and pressure conditions in the sputtering process. The thickness of the Pt films was 100 nm at various sputtering times. A higher deposition temperature resulted in larger grain sizes as shown in Fig. 2a–c. The maximum deposition temperature was 400 °C because of the limitations of the equipment. Therefore, the Pt layer with the largest grain size as shown in Fig. 2d was obtained from the outside source, which was deposited by DC sputtering at room temperature with the same thickness as the other Pt thin films (100 nm Pt/ 10 nm Ti) as a reference to see the tendency and make a clearer observation of the grain size effects. The grain size and size distribution of each sample were extracted using the ImageJ program [24]. The Pt thin films were deposited at room temperature, 200 °C, 400 °C, and the outsourced sample showed modal grain sizes of 17, 27, 37, and 55 nm, respectively. The TECs of face-centered cubic metallic thin films such as Ag and Cu are reported to increase with increasing grain size because of the fewer grain boundaries with larger grain sizes as the grain boundary absorbs thermal extension [25]. Hence, Pt thin films, which are in the face-centered cubic metallic group, are expected to reveal the same tendency as Ag and Cu. Different TECs of Pt thin films could induce the different stresses on the HZO film deposited during the annealing process. This means that a smaller Pt grain size could impart more tensile in-plane stress to the HZO thin film. The ferroelectric properties of HZO films would be expected to differ according to the Pt grain sizes.

**Fig. 3:**
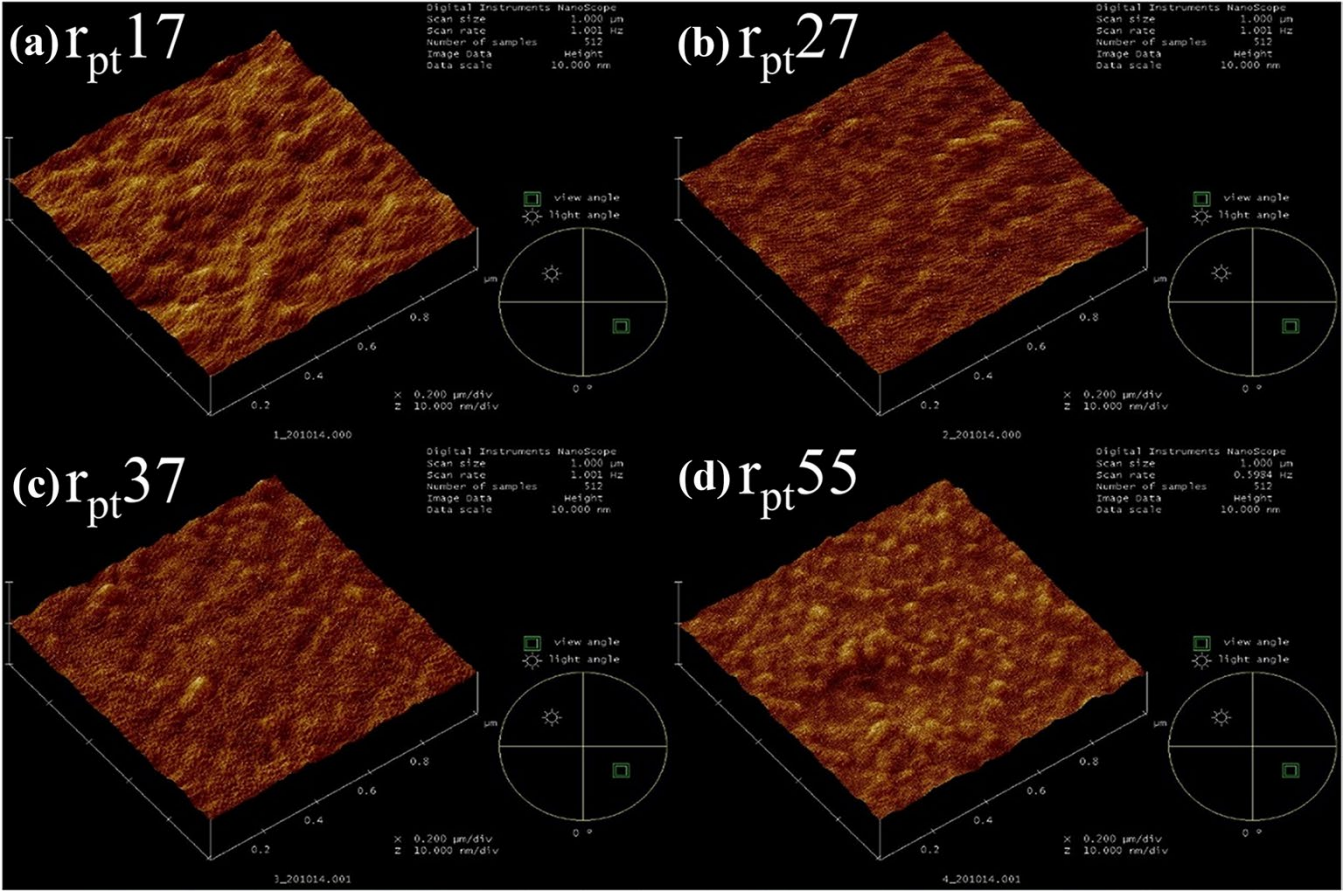
AFM images of HZO surfaces on various Pt substrates. The information in the inside panels includes scan size, scan rate, and data scale. The surface root-mean-square roughness (R_RMS_) of the HZO films was approximately 0.5 nm for all samples

The thickness of all the HZO thin films after post-deposition annealing is 30 nm. The surface and grain size of HZO thin films were extracted from the AFM images in Fig. 3 by the Gwyddion program with the mark by watershed [26]. The HZO samples were then marked according to the Pt grain size r_Pt_17, r_Pt_27, r_Pt_37, and r_Pt_55 as the Pt grain sizes from the SEM images. Larger HZO grains are induced as the Pt grain size of the bottom electrode increases. The grain sizes of HZO films were 4.8, 7.1, 9.5, and 10 nm, respectively. The surface root-mean-square roughness (R_RMS_) of the HZO films was approximately 0.5 nm for all samples. Similar R_RMS_ values suggest that the grain size of the bottom electrode affects the in-plane growth process of HZO layers.

**Fig. 4:**
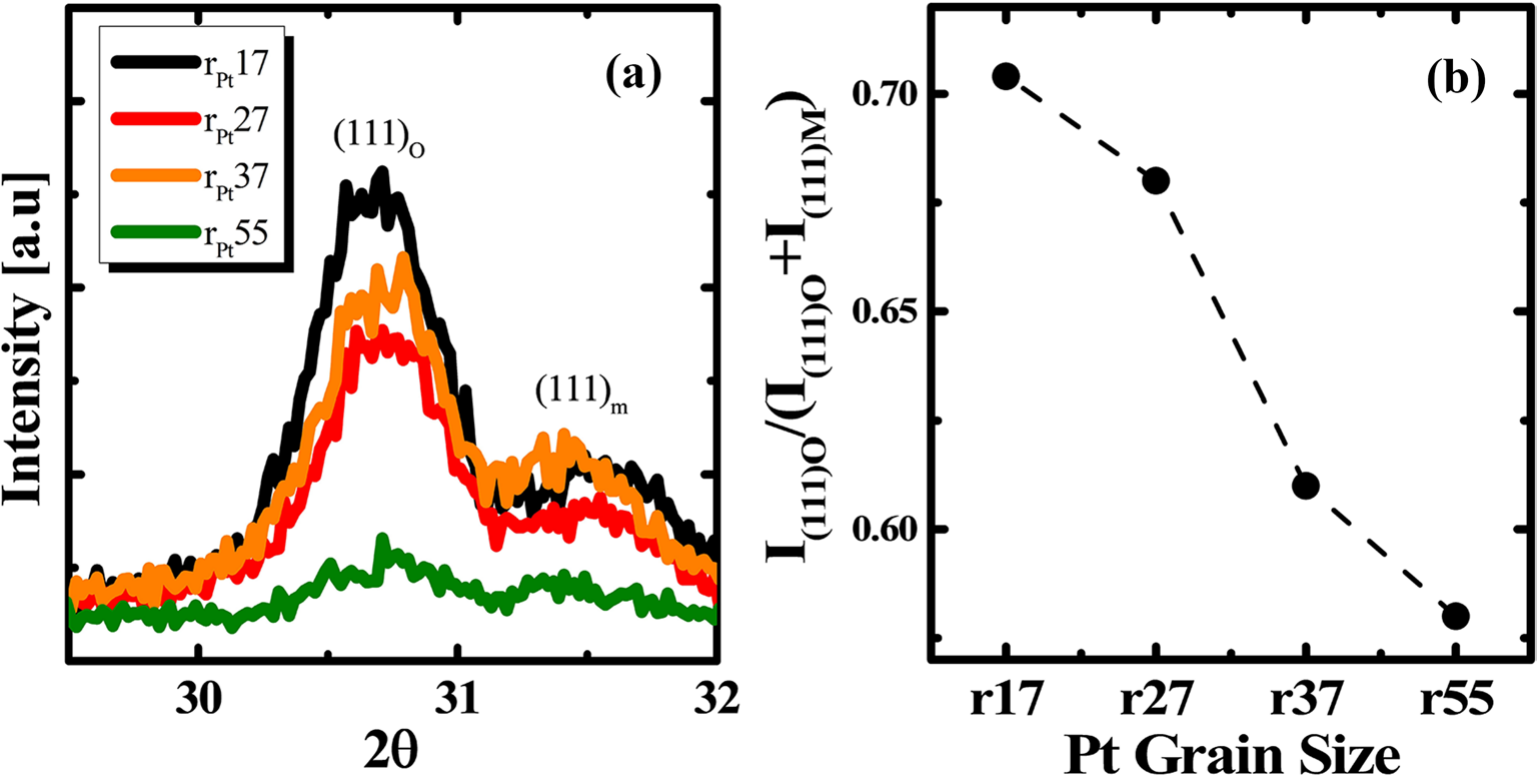
**a** GIXRD pattern and **b** fraction of the orthorhombic phase of HZO on various grain size Pt layers

The crystalline phases of the HZO thin films were analyzed by GIXRD as shown in Fig. 4. The GIXRD patterns had various peaks representing orthorhombic, tetragonal, and monoclinic phases after high-temperature post-deposition annealing. Precise quantitative phase composition in the HZO thin films is challenging because of peak overlap. The 29–32° 2θ range is commonly used to distinguish non-centrosymmetric and centrosymmetric phases. The (111) orthorhombic or (101) tetragonal phase was observed at approximately 30.7°. Another peak at approximately 31.5° was assigned to the (111) monoclinic phase. The intensity of both peaks decreased gradually with increasing Pt grain size, as shown in Fig. 4 a), suggesting a smaller orthorhombic phase composition. As mentioned in earlier reports, the orthorhombic phase is responsible for the ferroelectric properties [27]. The fraction (F) of the orthorhombic phase in the HZO polycrystalline structure is defined from the intensity of the peaks in the GIXRD results, $F = \frac{{I_{(111)O} }}{{I_{(111)O} + I_{(111)M} }}$, where I(111)_O_ and I(111)_M_ are the diffraction intensities of the peaks at 30.7° and 31.5°. Figure 4 b) presents the fractions showing a decrease in the orthorhombic phase with increasing Pt grain size. This suggests that the smaller Pt grain size produces more favorable stress for a transformation to the orthorhombic phase.

**Fig. 5:**
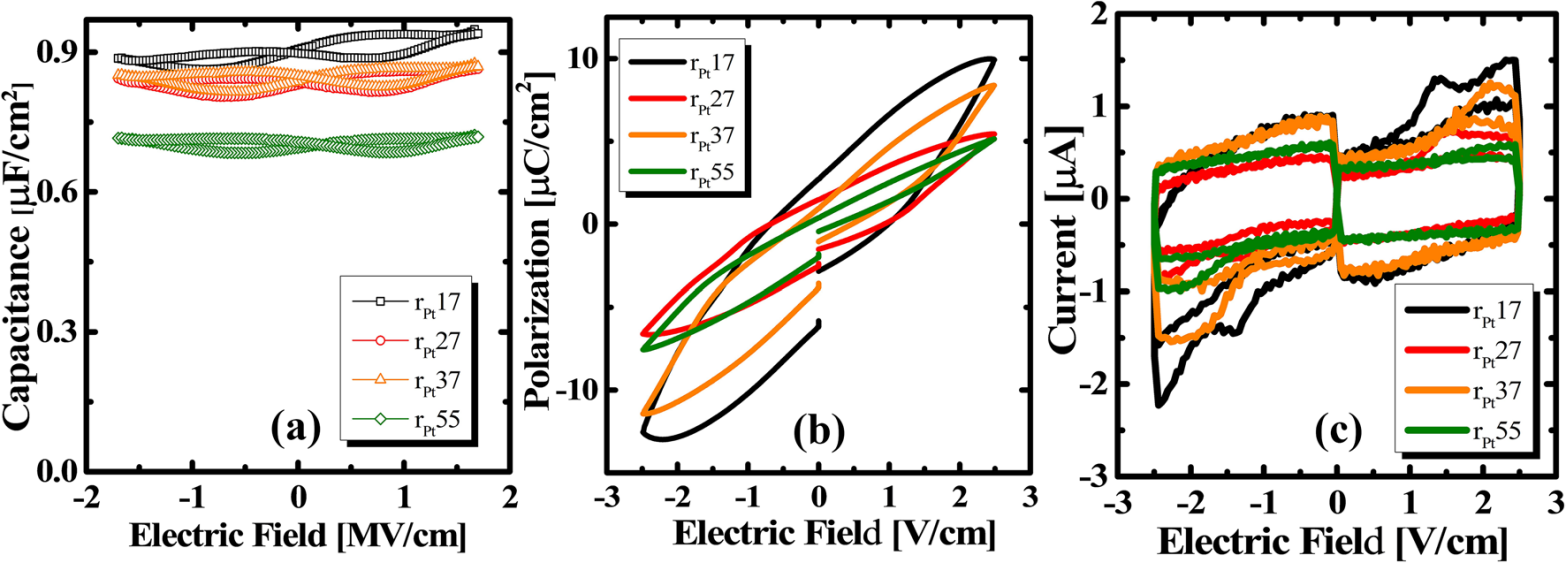
**a** C-E, **b** P-E, and **c** PUND performances of HZO MFM with various Pt grain sizes for bottom electrodes

The electrical properties of the MFM capacitors were correlated with the phase composition of each HZO film as shown in Fig. 5a, and b which show the C-E and P-E plots. The electrical properties of the HZO thin films differed from the size of grains of the Pt bottom electrodes. All MFM capacitors with different Pt bottom electrodes exhibited butterfly loops with two peaks in the C-E result, as shown in Fig. 5a at 100 kHz. The capacitance, however, tended to decrease with increasing Pt grain size, even though the leakage currents were similar. The estimated dielectric constants from capacitance results were 32.2, 29.5, 29.7, and 29.2 for the r_Pt_17, r_Pt_27, r_Pt_37, and r_Pt_55 samples, respectively. The dielectric constant is an average of the amount of each phase inside the HZO layers. Since the higher-symmetry crystalline phase as the cubic or tetragonal phase has a larger dielectric constant than the monoclinic phase (≈40–50, 32–40, and 17–20) [28, 29], the r_Pt_17 sample would have a more orthorhombic phase in the HZO thin-film, confirming the GIXRD results.

Figure 5b shows the featured shape of polarization in the P-E curves of HZO thin films, which are deposited on different Pt substrates. The hysteresis is measured under a triangular waveform with a 100 kHz frequency. The coercive electrical fields are approximately 2 MV/cm for all samples. The remnant polarization (2Pr) was approximately 7 μC/cm^2^ for r_Pt_17 and 2 μC/cm^2^ for r_Pt_55, showing a decrease with increasing Pt grain size, which agrees with the C-E tendency. The reduction observed in the P-E and C-E can be explained by the lower portion of the orthorhombic phase with increasing Pt grain size. The antiferroelectric phase increases when the grain size enlarges, as in the P-E curve is also the evidence for the grain size effect. As mentioned above, the small Pt grain size with a lower TEC induces a higher transformation for the orthorhombic phase, resulting in higher ferroelectric properties of the HZO thin film. These results correspond to other studies that used various substrates for different TEC values. Owing to the small grain size of the Pt layer, the TEC was also reduced, causing favorable in-plane tensile stress for the ferroelectric phase transformation. With different materials as substrates, the contrast was considerably high because of the large distance between the TECs. In addition, the small HZO grain size also expresses the greater ferroelectric properties as reported elsewhere [30, 31]. The grain size change leads to the various TECs and results in the change of the ferroelectric properties. For the ALD HZO thin film, they would still be affected by the bottom Pt grain size as the CSD HZO thin films, but the magnitude of the change of ferroelectricity would be different due to the various microstructure.

## Conclusions

HZO thin films were fabricated on Pt layers with various grain sizes to gain a better understanding of phase transformation. The smaller grain size of the Pt substrate leads to more transformation to the orthorhombic phase. This is explained by the lower TEC of the Pt substrate, which increases the in-plane tensile stress. Deposition at room temperature, r_Pt_17, which produced the smallest grain size for Pt, resulted in the largest polarization properties in this experiment. This result can be extended to other bottom electrode materials to enhance the ferroelectric properties.

## Data Availability

Not applicable.

## Abbreviations

HZO: Hafnium zirconium oxide
CSD: Chemical solution deposition
TEC: Thermal expansion coefficient
P-E: Polarization-electrical field
C-E: Capacitance-electrical field
DC: Direct current
RT: Room temperature
MFM: Metal-ferroelectric-metal
SEM: Scanning electron microscopy
AFM: Atomic force microscopy
GIXRD: Grazing incidence X-ray diffraction

## References

[1] Ni K, Sharma P, Zhang J, Jerry M, Smith JA, Tapily K, Clark R, Mahapatra S, Datta S (2018). IEEE Trans. Electron Devices.

[2] T. Francois, L. Grenouillet, J. Coignus, P. Blaise, C. Carabasse, N. Vaxelaire, T. Magis, and F. Aussenac, V. Loup, C. Pellissier, S. Slesazeck, V. Havel, C. Richter, A. Makosiej, B. Giraud, E. T. Breyer, M. Materano, P. Chiquet, M. Bocquet, E. Nowak, U. Schroeder, F. Gaillard, 2019 IEEE International Electron Devices Meeting (IEDM), 2019, pp. 15.7.1-15.7.4, 10.1109/IEDM19573.2019.8993485.

[3] S. Fujii, Y. Kamimuta, T. Ino, Y. Nakasaki, R. Takaishi, and M. Saitoh, 2016 IEEE Symposium on VLSI Technology, 1-2.

[4] C. Mart, S. Abdulazhanov, M. Czernohorsky, T. Kampfe, D. Lehninger, K. Falidas, S. Eslinger, K. Kuhnel, S. Oehler, M. Rudolph, M. Wiatr, S. Kolodinski, R. Seidel, W. Weinreich, and L. M. Eng, 2020 IEEE International Electron Devices Meeting (IEDM), 2020, pp. 26.3.1-26.3.4, 10.1109/IEDM13553.2020.9371967.

[5] Mart C, Kämpfe T, Czernohorsky M, Eßlinger S, Kolodinski S, Wiatr M, Weinreich W, Eng LM (2020). Appl. Phys. Lett..

[6] Di. Das, V. Gaddam, and S. Jeon, IEEE Electron Device Letters, vol. 42, no. 3, pp. 331-334, March 2021, 10.1109/LED.2021.3055140.

[7] Sang Xiahan, Grimley Everett D., Niu Changning, Irving Douglas L., LeBeau James M. (2015). On the structural origins of ferroelectricity in HfO2 thin films. Applied Physics Letters.

[8] Müller J, Böscke TS, Schröder U, Mueller S, Bräuhaus D, Böttger U, Frey L, Mikolajick T (2012). Nano Lett..

[9] Kim KD, Park MH, Kim HJ, Kim YJ, Moon T, Lee YH, Hyun SD, Gwon T, Hwang CS (2016). J. Mater. Chem. C.

[10] Materlik R, Kunneth C, Kersch A (2015). J. Appl. Phys..

[11] Yurchuk E, Müller J, Knebel S, Sundqvist J, Graham AP, Melde T, Schröder U, Mikolajick T (2013). Thin Solid Films.

[12] Fan Z, Chen J, Wang J (2016). J. Adv. Dielectr..

[13] Starschich S, Boettger U (2017). J. Mater. Chem. C.

[14] Böscke TS, Müller J, Bräuhaus D, Schröder U, Böttger U (2011). Appl. Phys. Lett..

[15] M. H. Park, H. J. Kim, Y. J. Kim, W. Lee, T. Moon, K. Do Kim, and C. S. Hwang, Appl. Phys. Lett. 105, 0 (2014). 10.1063/1.4893376.

[16] Li T, Zhang N, Sun Z, Xie C, Ye M, Mazumdar S, Shu L, Wang Y, Wang D, Chen L, Ke S, Huang H (2018). J. Mater. Chem. C.

[17] Lee YH, Kim HJ, Moon T, Do Kim K, Hyun SD, Park HW, Bin Lee Y, Park MH, Hwang CS (2017). Nanotechnology.

[18] Starschich S, Griesche D, Schneller T, Waser R, Böttger U (2014). Appl. Phys. Lett..

[19] Starschich S, Schenk T, Schroeder U, Boettger U (2017). Appl. Phys. Lett..

[20] Nakayama S, Funakubo H, Uchida H (2018). Jpn. J. Appl. Phys..

[21] Starschich S, Griesche D, Schneller T, Böttger U (2015). ECS J. Solid State Sci. Technol..

[22] Goh Y, Jeon S (2018). Nanotechnology.

[23] Shiraishi T, Katayama K, Yokouchi T, Shimizu T, Oikawa T, Sakata O, Uchida H, Imai Y, Kiguchi T, Konno TJ, Funakubo H (2016). Thing. Appl. Phys. Lett..

[24] Schneider CA, Rasband WS, Eliceiri KW (2012). Nat. Methods.

[25] Hwang S, Kim Y (2011). J. Nanosci. Nanotechnol..

[26] Nečas D, Klapetek P (2012). Cent. Eur. J. Phys..

[27] Lyu J, Fina I, Solanas R, Fontcuberta J, Sánchez F (2019). ArXiv.

[28] Hyuk Park M, Joon Kim H, Jin Kim Y, Lee W, Moon T, Seong Hwang C (2013). Think. Appl. Phys. Lett..

[29] Hoffmann M, Schroeder U, Schenk T, Shimizu T, Funakubo H, Sakata O, Pohl D, Drescher M, Adelmann C, Materlik R, Kersch A, Mikolajick T (2015). J. Appl. Phys..

[30] H. J. Kim, M. H. Park, Y. J. Kim, Y. H. Lee, W. Jeon, T. Gwon, T. Moon, K. Do Kim, and C. S. Hwang, Appl. Phys. Lett. 105, 192903 (2014). 10.1063/1.4902072.

[31] Kim BS, Hyun SD, Moon T, Do Kim K, Lee YH, Park HW, Bin Lee Y, Roh J, Kim BY, Kim HH, Park MH, Hwang CS (2020). Nanoscale Res. Lett..

